# Effects of pressure garments of varying designs on upper extremity sensorimotor functions and quality of life after stroke: Study protocol for a multicenter, double-blind, prospective randomized controlled trial

**DOI:** 10.1371/journal.pone.0326680

**Published:** 2025-06-23

**Authors:** Zhenkun Xu, Siaw Chui Chai, Shin Ying Chu, Kuicheng Li

**Affiliations:** 1 Center for Rehabilitation and Special Needs Studies, Faculty of Health Sciences, Universiti Kebangsaan Malaysia, Kuala Lumpur, Malaysia; 2 Center for Health Ageing and Wellness, Faculty of Health Sciences, Universiti Kebangsaan Malaysia, Kuala Lumpur, Malaysia; 3 School of Rehabilitation Medicine, Shandong Second Medical University, Weifang, China; The University of Manchester, UNITED KINGDOM OF GREAT BRITAIN AND NORTHERN IRELAND

## Abstract

**Rationale:**

Stroke often results in extensive neurological damage, leading to a wide range of rehabilitation needs and challenges, with upper extremity dysfunction being particularly prevalent. Although pressure garments have been used in the rehabilitation of children with cerebral palsy to reduce muscle tone, their therapeutic effects have not been thoroughly investigated in the field of stroke.

**Aims:**

To determine the effects of pressure garments with varying designs on stroke patients’ sensorimotor function and quality of life.

**Sample size estimate:**

A total of 165 participants is required (55/group) with an effect size of 0.125, power of 0.80, alpha level of 0.05, and adjusted for a dropout rate of 20%.

**Methods and design:**

This is a multicenter, double-blind, prospective randomized controlled, three-group trial. At three hospitals in Shandong, China, 165 patients within 1−12 months of stroke are randomly assigned (1:1:1) to receive Dorsal-Double-layered 10% circumferential reduction (DD-10, intervention), Single-layered 10% circumferential reduction (S-10, intervention), or Single-layered 0% circumferential reduction (S-0, placebo) pressure garments. Pressure garments are worn for 3 hours in the morning, 3 hours in the afternoon, and 8 hours at night daily for 8 weeks. During the first 4 weeks, patients also receive 30-min occupational therapy sessions.

**Study outcomes:**

The primary outcome is the Fugl-Meyer Assessment of Upper Extremity to assess motor control. Secondary outcomes are the Box and Block Test (BBT) for assessing dexterity, Modified Ashworth Scale (MAS) for assessing muscle tone, Visual Analogue Scale for assessing pain, Disabilities of Arm, Shoulder, and Hand (DASH) for assessing self-perceived upper extremity function, and 36 Item Short Health Survey (SF-36) for assessing quality of life. Measurements are taken at Time 1(Baseline), Time 2 (Week 4), and Time 3 (Week 8).

**Discussion:**

The expected outcome of this study is that it can determine the design of pressure garments best suited to improve sensorimotor function and the quality of life of stroke patients. It can also extend the clinical value of pressure garments and help healthcare professionals make more targeted treatment choices for stroke patients.

**Trial registration:**

ClinicalTrials.gov Identifier: NCT06587308.

## Introduction

Stroke is a challenging neurological disorder worldwide and remains the second leading cause of death and disability in the world [1]. In China, the estimated prevalence, incidence, and mortality rate of stroke among adults aged 40 years or older in 2020 were 2.6%, 505.2/100,000 person-years, and 343.4/100,000 person-years, respectively [2]. Stroke often results in extensive neurological damage, leading to a wide range of rehabilitation needs and challenges, with upper extremity dysfunction being prevalent [3–5]. This dysfunction includes impaired sensorimotor function with abnormal muscle tone, pain, poor dexterity, etc., eventually affecting quality of life.

A pressure garment is an external tool made of elastic material, commonly Lycra, that applies pressure to improve swelling, inhibit scarring, and enhance motor performance [6,7]. In sports, moderate pressure may enhance blood circulation and reduce poor venous return and muscle fatigue, increasing comfort and athletic performance [8]. Concerning muscle tone, pressure garments are thought to provide neutral heat by reducing spasms through prolonged stretching and skin irritation from tight skin contact [9]. By reducing the high muscle tone of the spastic muscles, the application of pressure to the damaged limb allows for better control of the antagonistic muscles [7]. The uniform pressure applied by pressure garments may also modulate sensory input and improve joint position sense and body awareness by stimulating mechanoreceptors [10]. Although it is used in the rehabilitation of children with cerebral palsy for the reduction of muscle tone [11], its therapeutic effects have not been fully confirmed in the field of stroke. Cerebral palsy and stroke, although they may exhibit the characteristics of elevated spasticity and dyskinesia, create similar problems with upper extremity function; application of evidence gathered from cerebral palsy to stroke rehabilitation requires cautious attention. Since cerebral palsy involves still-developing muscles, stroke involves fully matured muscles [12], a specific research on stroke is essential to ensure safe and effective pressure garment applications in stroke rehabilitation.

The effects of pressure garments on upper extremity functions after stroke are limited and controversial. Gracies et al. [13] designed a crossover trial that included a total of 16 patients (36–85 years old; > 3 weeks of stroke) with hemiparesis and upper extremity spasticity and found that wearing a Lycra garment for 3 hours improved wrist posture and reduced wrist and finger flexor spasticity. Conversely, Ooi et al. (2020) [14] found no difference in arm spasticity and function after 6 hours per day, 6-week pressure garment applications. However, verbal feedback informed that the pressure garment allows greater finger extension to facilitate grasping and releasing movements. According to Ooi et al. (2020) [14], this failure is likely due to insufficient wearing time, inappropriate design, and small sample size. For future studies, besides using larger sample size, they also suggested to add an extra Lycra on the pressure garment’s dorsal aspect, extending the coverage proximally beyond the elbow joint, and prolonging its wearing time to improve the extension force [14]. According to this recommendation [14], our study is designed to gather more substantiate evidence to support pressure garment clinical usage in stroke rehabilitation.

### Objectives

The primary objective of this study is to determine the effects of pressure garments with varying designs (i.e., Dorsal-Double-layered 10% circumferential reduction; Single-layered 10% circumferential reduction; and Single-layered 0% circumferential reduction) on sensorimotor function, including motor control, dexterity, muscle tone, pain, self-perceived upper extremity function and quality of life in stroke patients after 8 weeks of intervention.

### Hypotheses

There is a difference between the effects of DD-10, S-10, & S-0 PG on motor control, dexterity, muscle tone, pain, self-perceived upper extremity function as measured by Fugl-Meyer Assessment of Upper Extremity [15], Box and Block Test (BBT), Modified Ashworth Scale (MAS), Visual Analogue Scale, Disabilities of Arm, Shoulder, and Hand (DASH), and 36 Item Short Health Survey (SF-36), respectively, among stroke patients during 8 weeks of application. Having an extra piece of fabric on the dorsal side, DD-10 is expected to provide stronger power of extension to the patient’s hand, wrist, and elbow, thus helping open the flexed upper extremity for better sensorimotor performance.

## Method

### Research design

**Fig 1:**
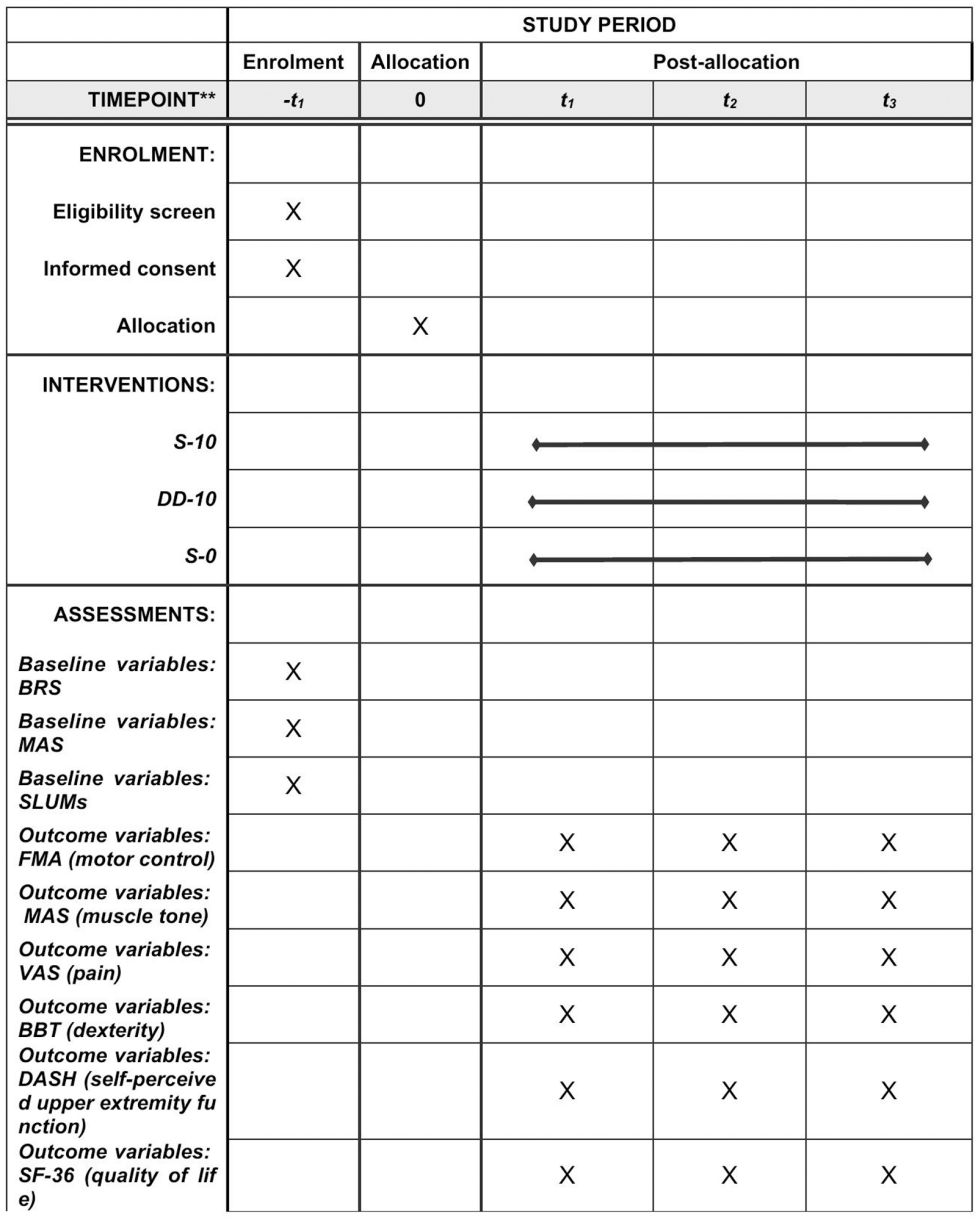
Study schedule.

**Fig 2:**
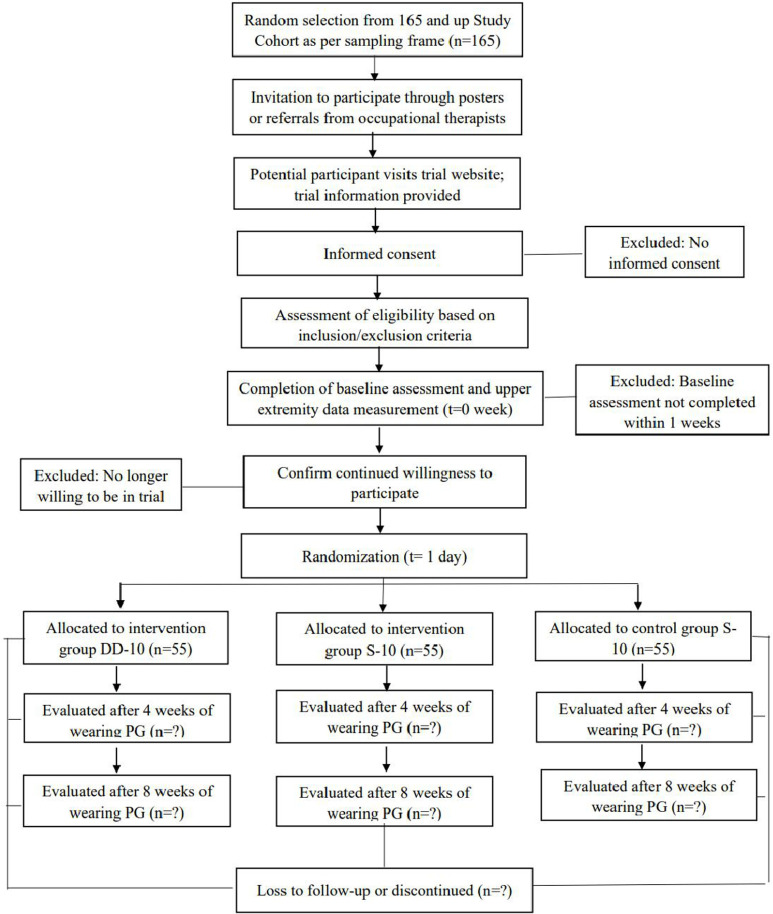
Study flowchart.

This is a multicenter, double-blind, prospective randomized controlled, three-group trial, designed according to the criteria of the SPIRIT 2013 statement. The checklist for this study is detailed in S1 File. The study protocol is detailed in S2 File. The study schedule and flowchart are shown in Figs 1 and 2. The first participant was recruited on October 08, 2024. Participant recruitment and data collection are expected to be completed in June 2026 and August 2026, respectively. The study results are expected to be submitted for publication as soon as we complete the data analysis and result writing.

### Participants: Recruitment and eligibility criteria

The inclusion criteria are stroke patients aged 18–80 years old with 1–2 months of first onset ischemic or hemorrhagic stroke that resulting in hemiparesis of a limb with Brunnstrom Stage of Recovery (BSR) of 3, 4 or 5, Modified Ashworth Scale (MAS) ≤level 2 for elbow, wrist, and hand muscle tone (levels 3 and 4 patients may have significant difficulty in wearing pressure garments due to spasticity, posing safety concerns include skin macerations, sensation and circulation problems, especially around the joints), and able to understand instruction. Those with serious cardiovascular or respiratory diseases, skin lesions, infections or other skin problems, serious circulatory problems such as deep vein thrombosis, allergies or significant discomfort to pressure garment materials, upper extremity fractures or severe joint problems, and severe cognitive impairment with Saint Louis University Mental Status (SLUMs) scores ≤27 (above high school level of education) or 25 (below high school level of education) are excluded [16].

Participants are inpatients from three hospitals in Weifang, China, i.e., the Affiliated Hospital of Shandong Second Medical University, Weifang Municipal People’s Hospital, Weifang Hospital of Traditional Chinese Medicine.

### Sampling size

Sample size was calculated using G*Power 3.1. This study intends to use a mixed model ANOVA to compare differences between the effects of DD-10, S-10, and S-0 pressure garments. Due to the lack of prior studies on this novel intervention, for exploratory purposes, we estimated that a small to medium effect size, f = 0.125, would be sufficient to yield significant outcomes. With a power = 0.8, and α error = 0.05, the total sample size needed is 132. With 44 patients in each group and considering a 20% dropout rate, the use of the formula N = n/(1-d), that is, 44/ (1-0.2), yields 55 patients in each group. Hence, the final sample size are 165 patients.

### Randomization

**Fig 3:**
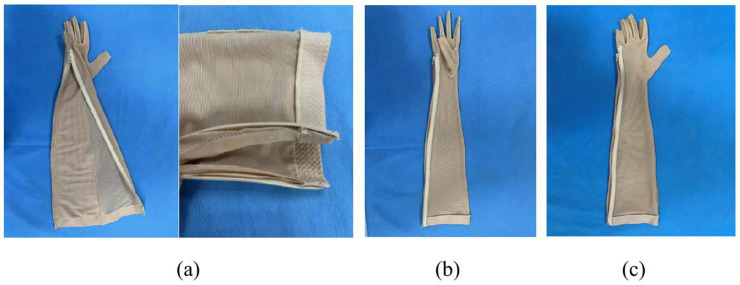
Pressure garment.

A total of 165 sequences are generated using a computerized software SPSS 26. These sequences contain equal numbers of 1 (single-layered 10% circumferential reduction, S-10, intervention), 2 (dorsal-double-layered 10% circumferential reduction, DD-10, intervention), and 3 (single-layered 0% circumferential reduction, S-0, placebo), stored electronically by the first author (ZKX) who is not involved in conducting the interventions or assessments. Participants are randomly assigned (1:1:1) to receive DD-10, S-10, or S-0 pressure garment, as shown in Fig 3. The overall approach to the DD-10 pressure garment is the same as that for the S-10 pressure garment, with the main difference being that the dorsal side is simply secured using two pieces of fabrics of the same size and then sewn together as a whole. The reduction factor is calculated using a standard % reduction on all bodies, regardless of size/fabric or Laplace’s Law, and the fabric tension profile to calculate specific reduction factors. The fabric used in this study is made and supplied by Klarity, China.

### Blinding

Therapist A (an assessor, n = 3), Therapist B (a treating therapist, n = 6), and participants are blinded. At Week 8, the success of blinding assessment using the statistical method of James’ Blinding Index (BI) [17] is conducted by asking participants to indicate the group that they belonged to, i.e., either DD-10, S-10, S-0 or don’t know. Therapist A is also asked to indicate the same question regarding group location of the participant that they had assessed. Those who do not answer the questions are removed from the blinding assessment.

### Research team

This study involves six therapists, typically two therapists [Therapists A (an assessor) and Therapist B (a treating therapist)] from each data collection site (i.e., the Affiliated Hospital of Shandong Second Medical University, Weifang Municipal People’s Hospital, Weifang Hospital of Traditional Chinese Medicine), respectively.

### Informed consent, registration, baseline assessment and group allocation

The study flow is explained to the selected participants upon confirmation of their eligibility for the study. An information sheet containing general information, purpose, procedures, risks and benefits, as well as data confidentiality, are provided and explained to each participant. Participants need to signed the informed consent form before participating in the study. After this, baseline assessment and collection of demographic data for each participant are performed by Therapist A. Subsequently, participants are allocated into either the S-10, DD-10, S-0 groups by the first author (ZKX) through simple randomization with allocation concealment (Fig 2).

### Intervention

At Time 1 (Baseline), eligible participants are given a custom fabricated DD-10, S-10, or S-0 pressure garment to be worn for 30 minutes. Participants are formally enrolled in the study if no allergy, edema, pain or any other discomfort are observed. The actual wearing schedule is 14 hours, that is, 3 hours in the morning, 3 hours in the afternoon, and 8 hours in the evening each day for 8 weeks. Each participant is given a Pressure Garment Application Form to complete. The participant is required to return the form to the researcher during the Time 2 (Week 4) assessment, and at the same time, a new form is given to the participant for completion and collection by the researcher during the Time 3 (Week 8) assessment. Therapist B (a treating therapist) will constantly monitor the condition of the pressure garment to make sure it stays tightly fit throughout the study. Depending on the garment’s condition, torn or loosed pressure garment are repaired or replaced using a new pair. For the first 4 weeks, participants also receive occupational therapy conducted by Therapist B (a treating therapist) once a day for 30 minutes, 5 days a week. The sequence of therapy are: (1) 5-min warm-up activities, including upper extremity exercises, including arm swings, finger flexion and extension and wrist activities; (2) 10-min remedial activities, including building blocks, moving sticks, puzzles, twisting screws, threading beads; (3) 10-min activities of daily living training, including the use of simulated daily life scenarios (e.g., bathing, dressing, eating, etc.), use of assistive devices with step-by-step guidance to ensure participant’s safety and successful completion; and (4) 5-min relaxation activities, including deep breathing exercises, progressive muscle relaxation that focuses on stress-prone areas such as the neck, shoulders and back, static stretches and other exercises to help relax the body. Participants are not required to attend occupational therapy for the remaining 4 weeks.

As stated in the information sheet, the potential risks of wearing the pressure garment include allergic reaction, circulatory problems, pain or loss of sensation in the affected upper extremity. Should these symptoms happen, participants must immediately remove the garment and inform the treating therapist.

### Primary outcome

#### Fugl-Meyer assessment.

Fugl-Meyer Assessment is used to assess the rehabilitation process of stroke patients and provide insight into the participant’s sensorimotor function and level of life activities [18]. It has two main domains, the upper extremity and lower extremity [19]; however, only the upper extremity domain is used in this study. This domain includes the range of motion of the shoulder, elbow, wrist, and fingers and elements such as grip strength and hand coordination. Scored between 0 and 66, higher scores indicating greater function. FMA is administered by Therapist A (an assessor) at Time 1 (Baseline), Time 2 (Week 4), and Time 3 (Week 8). At weeks 4 and 8, the administration is 30 minutes after pressure garment removal.

### Secondary outcomes

Secondary outcomes including Modified Ashworth Scale (MAS), Visual Analogue Scale, Box & Block Test (BBT), Disabilities of Arm, Shoulder and Hand (DASH) Outcome Measure, and 36-Item Short Form Health Survey (SF-36) are administered by Therapist A at Time 1 (Baseline), Time 2 (Week 4), and Time 3 (Week 8) to assess muscle tone, pain, dexterity, self-perceived upper extremity function, and quality of life, respectively. The administration of assessments at week 4 and week 8 are: MAS – immediately after pressure garment removal; DASH and SF-36 – within 30 minutes of pressure garment removal; and VAS and BBT – 30 minutes after pressure garment removal.

#### Modified Ashworth Scale (MAS).

The MAS is used to assess muscle tone or spasticity, especially among stroke patients [20]. It uses a scale of 0–4, where 0 indicates no spasticity and 4 indicates severe spasticity, with each scale being clearly described.

#### Visual Analogue Scale.

The VAS a subjective measurement scaling tool that is commonly used to assess an participant’s sensations or feelings on specific dimensions [21]. A 10-cm pain scale with one end of the scale represents no pain and the other end represents unbearable pain. Subjective perception of pain is obtained by drawing a vertical line between these two endpoints.

#### Box and Block Test (BBT).

The BBT is used to assess upper extremity sensorimotor function, particularly coordination, dexterity, speed of movement, and fine hand movements [22]. Participants are required to move a set of cubes from one side of a box separated into two equal-sized compartments to the other side, with a divider to prevent the cubes from falling out. It is assessed based on the number of cubes successfully transfers in one minute [22].

#### Disabilities of Arm, Shoulder and Hand (DASH) outcome measure.

DASH is a 30-item outcome measure used to measure upper extremity related functional problems, including life activities, symptoms, social roles and psychological aspects. Scores between 0 and 100, with higher scores indicating more severe disability [23].

#### 36-Item Short Form Health Survey (SF-36).

SF-36 is a 36-item questionnaire designed to provide insight into an individual’s health status and quality of life related to the physical, psychological, and social dimensions of the patients. Each dimension is converted into a score of 100 points by assigning weights to the entries according to the degree to which they affect the quality of life. The level of the score directly reflects health status, with higher scores indicating better functional status and quality of life in this area [24].

### Statistical analysis

Intention-to-treat (ITT) analysis is used to maintain random allocation of study participants [25] and prevent shedding bias when shedding participants [26]. Imputation methods, such as Last Observation Carried Forward (LOCF) [27], mean imputation [28], or multiple imputation [29] may be used depending on the requirements of the data. Frequencies, means, and medians are used to describe the demographic data [30]. A mixed ANOVA is used to compare the outcome measures with level of significance set to p < 0.05.

### Monitoring, ethics and data monitoring body

This study has obtained ethical approval from the Research Ethics Committee of Universiti Kebangsaan Malaysia (JEP-2024–582), the Affiliated Hospital of Shandong Second Medical University, Weifang Municipal People’s Hospital, Weifang Hospital of Traditional Chinese Medicine. All participants are voluntary and all data will be kept strictly confidential to ensure anonymity of the participants’ identity. The study results may be published in aggregate form but will not contain any identifiable personal information. The Ethics Research Committee of the leading organization and three trial sites will ensure that the trial is adhering to ethical guidelines. All sites are required to report minor and serious adverse events occurring through the course of the trial. The trial results will be reported in accordance with the Consolidated Standards of Reporting Trials (CONSORT) guidelines. The manuscript detailing the trial will be submitted to a peer-reviewed international scientific journal for publication. Additionally, the findings may be presented at national conferences and scientific meetings.

## Results

This study was commenced on October 2024. Currently, the data collection process for protocol implementation is still ongoing.

## Discussion

This study investigates the effects of different designs of pressure garments on sensorimotor function and quality of life in stroke patients through a set of 8-week randomized controlled trial. The primary measure is FMA and secondary measures are MAS, VAS, BBT, DASH, SF-36, which are assessed at baseline, week 4, and week 8. To date, this is the first study that attempts to use dorsal double-layered pressure garments to improve the elicitation of extension force. This novel research is expected to support pressure garment application in improving sensorimotor function and quality of life of stroke patients, thus expanding the clinical value of pressure garments and allowing decision of more targeted intervention choices. Compared to published studies, this study uses longer pressure garment wearing duration and larger sample size to yield more impactful, reliable, and representative outcomes. In summary, there is a lack of large-scale trials of pressure garments in the stroke field. This large trial will provide evidence of the effects of different types of pressure garments on sensorimotor function and quality of life in stroke patients.

## Supporting information

S1 File SPIRIT checklist. (DOCX)

S2 File Study protocol. (PDF)
