# Supplementary material for: Effects of pressure garments of varying designs on upper extremity sensorimotor functions and quality of life after stroke: Study protocol for a multicenter, double-blind, prospective randomized controlled trial
Source: PLoS One. 2025 Jun 23;20(6):e0326680. doi: 10.1371/journal.pone.0326680 (PMC12184997; doi:10.1371/journal.pone.0326680)
Supplement: S2 File — (PDF) [file pone.0326680.s002.pdf]

Study Protocol

EFFECTS OF PRESSURE GARMENTS OF VARYING DESIGNS ON UPPER EXTREMITY SENSORIMOTOR FUNCTIONS AND QUALITY OF LIFE AFTER STROKE: A MIXED-METHODS STUDY

Zhenkun Xu[1, 3], Siaw Chui Chai[*1,] Shin Ying Chu[2], Kuicheng Li[3]

[1]Center for Rehabilitation and Special Needs Studies, Faculty of Health Sciences, Universiti Kebangsaan Malaysia

[2]Center for Health Ageing and Wellness, Faculty of Health Sciences, Universiti Kebangsaan Malaysia

[3]School of Rehabilitation Medicine, Shandong Second Medical University

CONTENT

1.0 Introduction 4

2.0 Problem Statement 5

3.0 Research Significance 6

4.0 Research Questions 6

5.0 Research Objectives 7

6.0 Research Hypotheses 7

7.0 Conceptual Framework 8

8.0 Operational Definitions 9

9.0 Research Methodology 10

9.1 Multicentre, Double-Blind, Prospective Randomised Controlled Trial (Research Question 1; Specific Objective 1; Hypotheses 1 to 7) 10

9.1.1 Research Design 10

9.1.2 Participants 10

9.1.3 Research Location 12

9.1.4 Research Instruments 13

9.1.5 Data Collection 22

9.1.6 Data Analysis 27

9.1.7 SPIRIT Process 27

10.0 Reference 28

Appendix A Demographic Form (Participant) 33

Appendix B Fugl-Meyer Assessment 35

Appendix C Visual Analogue Scale 35

Appendix D1 Disabilities of the Arm, Shoulder and Hand 35

Appendix E Box and Block Test Result Form 35

Appendix F Modified Ashworth Scale 355

Appendix G SF-36 Questionnaire 355

Appendix H Brunnstrom Recovery Stage 355

Appendix I1 SLUMS Examination 36

Appendix J Pressure Garment Application Form 366

Appendix K Phase I Information Sheet 377

Appendix L Informed Consent Form 400
Appendix M Participant blinding questionnaire 422
Appendix N Therapist blinding questionnaire 433

1.0 Introduction

Stroke is a challenging neurological disorder worldwide and remains the second leading cause of death and disability in the world (Feigin et al. 2022). Currently, 16.9 million people worldwide suffer from stroke each year, with an incidence rate of 258/100,000 person-years (Béjot et al. 2016). The incidence of stroke in mainland China in 2020 is 505.2/100,000 person-years (Tu et al. 2023). In 2017, there were 1.12 million stroke cases in the European Union (Wafa et al. 2020). Strokes may cause extensive neurological damage, leading to a wide range of rehabilitation needs and challenges, with upper extremity dysfunction being particularly common (Raghavan 2015). This dysfunction can affect quality of life (Carod-Artal & Egido 2009).

A pressure garment is an external tool made of elastic material that applies pressure to improve swelling, inhibit scarring, and enhance motor performance (Anzarut et al. 2009). Because of its effect of reducing muscle tone, it is now also widely used in the rehabilitation of children with cerebral palsy (Gerard et al. 2022). However, in the field of stroke, the therapeutic effect of pressure garment has not yet been more fully investigated. A paper published by Gracies et al. (2000) mentions that pressure garment has an antispastic effect on the wrists and fingers of hemiplegic patients., thus, they can help patients with severe spasticity or painful swelling of the extremities. A randomised controlled trial by (Ooi et al. 2020) showed no difference between intervention group that received a pressure garment and control group that received no pressure garment in terms of arm spasticity and function in the early post-stroke period; however, verbal feedbacks provided by the intervention group patients were generally positive, especially pressure garment allowed greater finger extension, facilitating better grasp and release. The authors justified inadequate wearing time, inappropriate pressure compression related to design and length of the pressure garment, and small sample size as the primary reasons that resulted in these unfavourable outcomes. Therefore, their recommendation for future studies included focusing on improving the design of the pressure garment, alongside increasing wearing time and sample size.

Based on the given justifications and recommendations, it is salient that the

clinical applications of pressure garment after stroke are yet to be fully explored and valued. Therefore, an in-depth and comprehensive study using a mixed methods design is needed. This proposed study will involve using multicentre double-blind randomized controlled trial, photovoice, and semi-structured interview to determine the effects of different pressure garment on sensorimotor functions and quality of life among stroke patients in Weifang, Shandong Province, China. Findings of this study are expected to be of value for stroke rehabilitation, primarily in accelerating the rehabilitation process and optimizing stroke recovery. This study is expected to promote evidence-based practice, aligning with China governmental policy "Healthy China 2030". For Malaysia, this research is aligned with the "National Policy for Quality in Healthcare 2022-2026" that emphasizes of providing high quality healthcare that is safe, effective, and efficient to patients.

2.0 Problem Statement

Stroke can lead to sensorimotor dysfunction, impaired sensation, and an imbalance in muscle tone between the flexor and extensor systems, causing the elbow, wrist, and fingers to assume a flexed position (Hatem et al. 2016). These disturbances may result in an inability to grasp and release, impairing the ability to perform functional activities. Given that such limitations may lead to a reduced quality of life, effective upper extremity stroke rehabilitation is needed. Regaining upper extremity sensorimotor functions after stroke has always been a complex and challenging task. Patients often need multiple methods to facilitate recovery. Pressure garment, although clinically has been used to reduce spasticity in the rehabilitation of children with cerebral palsy, it is only be used very limitedly in stroke rehabilitation. This is basically due to limited evidence-based research to support positive effects of pressure garment for stroke recovery. Particularly, previous studies have sample sizes, inappropriate garment design and wearing duration. Therefore, by referencing Ooi et al.'s (2020), there are a few issues that need to answered. Typically, pertaining to effectiveness of different garment designs (varying in terms of fabric layer and circumferential reduction),

amount of the sensorimotor functional changes that can be detected in the upper extremity, alongside changes of quality of life after pressure garment application. It is also important to explore the perceptions of stroke patients on pressure garment wearing experience, needs, challenges, and satisfaction as well as perceptions of treating therapists regarding the clinical values of pressure garment in the treatment for stroke.

3.0 Research Significance

Aligning with China's governmental policy "Healthy China 2030" and Malaysia's "National Policy for Quality in Healthcare 2022-2026" that emphasizes providing high quality healthcare that is safe, effective, and efficient to patients, this study is significant as it will promote stroke rehabilitation for the optimization of stroke recovery. By studying the effect of pressure garment on upper extremity dysfunction, healthcare professionals can make more targeted treatment choices and improve the chances of successful rehabilitation. Specifically, the success of improving patients' upper extremity dysfunction, followed by daily functioning and quality of life. This study will also expand the clinical values of pressure garment. If pressure garment is found to be effective in the rehabilitation of stroke patients, it can help to further expand the clinical values of pressure garment beyond its conventional usages, i.e., for scar and oedema management.

Most importantly, examining the effects of pressure garments empirically via combination of both quantitative and qualitative methodologies will improve the robustness of the study findings, in line with the need of implementing evidence-based practice.

4.0 Research Questions

General Research Question:

What are the effects of different finger-to-above-elbow (long glove) pressure garments with varying fabric layer and circumferential reduction on sensorimotor functions and quality of life among stroke patients during the 8 weeks of application,

and what are the perceptions of stroke patients and treating therapists on various issues relating to pressure garment application in stroke rehabilitation?

Specific Research Objectives:

To what extent that there is a difference between the effects of dorsal-double-layered 10% circumferential reduction (DD-10), single-layered 10% circumferential reduction (S-10), and single-layered no circumferential reduction (S-0) pressure garment on sensorimotor functions, including motor control, dexterity, muscle tone, pain, self-perceived upper extremity function, and quality of life among stroke patients during the 8 weeks of application (Time 1: Baseline, Time 2: Week 4, Time 3: Week 8)?

5.0 Research Objectives

General Research objectives:

To study the effects of different finger-to-above-elbow (long glove) pressure garment with varying fabric layer and circumferential reduction on sensorimotor functions and quality of life among stroke patients during the 8 weeks of application, alongside exploring the perceptions of stroke patients and treating therapists on various issues relating to pressure garment application in stroke rehabilitation.

Specific Research Objectives:

To determine the difference between the effects of dorsal-double-layered 10% circumferential reduction (DD-10), single-layered 10% circumferential reduction (S-10), and single-layered no circumferential reduction (S-0) pressure garment on sensorimotor functions including motor control, dexterity, muscle tone, pain, self-perceived upper limb function, and quality of life among stroke patients during the 8 weeks of application (Time 1: Baseline, Time 2: Week 4, Time 3: Week 8).

6.0 Research Hypotheses

1. There is a difference between the effects of DD-10, S-10, and S-0 pressure garment on motor control as measured by Fugl-Meyer Assessment of Upper Extremity

(Kaufman et al.) among stroke patients during 8 weeks of application.

2. There is a difference between the effects of DD-10, S-10, and S-0 pressure garment on dexterity as measured by Box and Block Test (BBT) among stroke patients during 8 weeks of application.

3. There is a difference between the effects of DD-10, S-10, and S-0 pressure garment on muscle tone as measured by Modified Ashworth Scale (MAS) among stroke patients during 8 weeks of application.

4. There is a difference between the effects of DD-10, S-10, and S-0 pressure garment on pain as measured by Visual Analogue Scale (Venckūnas et al.) among stroke patients during 8 weeks of application.

5. There is a difference between the effects of DD-10, S-10, and S-0 pressure garment on self-perceived upper extremity function as measured by Disabilities of Arm, Shoulder and Hand (DASH) among stroke patients during 8 weeks of application.

6. There is a difference between the effects of DD-10, S-10, and S-0 pressure garment on quality of life as measured by 36 Item Short Health Survey (SF-36) among stroke patients during 8 weeks of application.

7.0 Conceptual Framework

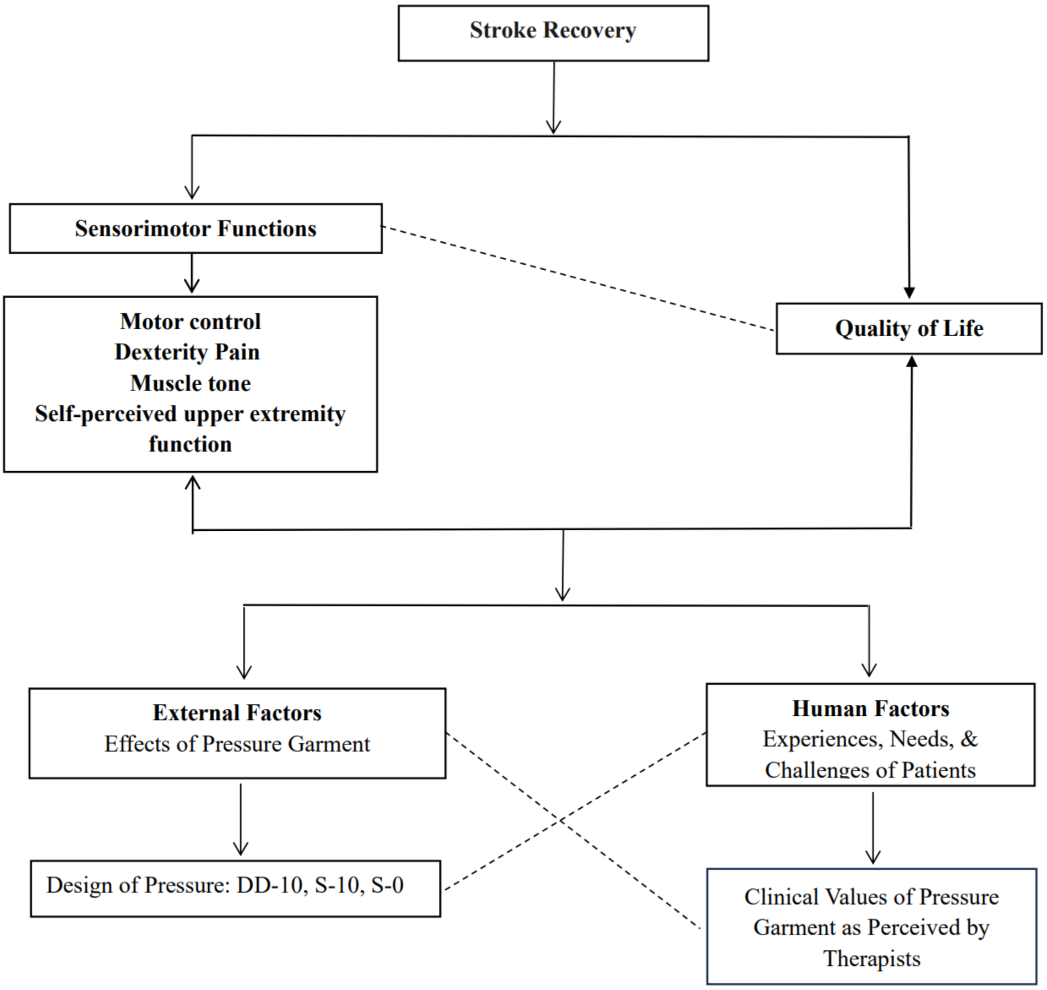

Figure 3. Conceptual Framework

The conceptual framework is guided using the ICF model, and the impacts of stroke are mainly reflected in two aspects, sensorimotor functions and quality of life. The sensorimotor functions specifically include motor control, dexterity, muscle tone, pain, self-perceived upper extremity function and the quality of life. External factors are the different types of pressure garments, i.e., DD-10, S-10, S-0. Human factors are the patient's experiences, needs and challenges, and the clinical value of pressure garment as perceived by the therapists. These external and human factors interact with each other.

8.0 Operational Definitions

Sensorimotor functions refer to the perception of sensory functions, including pain and motor functions, including motor control, dexterity, and self-perceived upper extremity function of a stroke patient as measuring by Visual Analogue Scale (Venckūnas et al.), Fugl-Meyer Assessment (Kaufman et al.) of upper extremity, Box and Block Test (BBT), Modified Ashworth Scale (MAS), and Disabilities of the Arm, Shoulder and Hand (DASH), respectively.

Quality of life refers to the ability of a stroke patient to perform various tasks in daily lives, including self-care, walking, socialization, and the negative impact of stroke on the patient's physical and mental health, including fatigue and mood disorders, reflected as the overall satisfaction with life that the stroke patient feels physically, mentally, and socially as measured by the 36-item Short Form Health Survey (SF-36).

9.0 Research Methodology

9.1 Multicentre, Double-Blind, Prospective Randomised Controlled Trial

(Research Question 1; Specific Objective 1; Hypotheses 1 to 7)

9.1.1 Research Design

Multicentre, double-blind, prospective randomised controlled trial will be used as it involves three research locations, blinding of participants and therapists, and prospective randomization of the participants into different intervention groups or placebo group.

9.1.2 Participants

Stroke patients who are admitted to a rehabilitation department of a hospital for rehabilitation services.

9.1.2.1 Inclusion and Exclusion Criteria

Inclusion criteria:

1. Between 18 and 80 years of age
2. Within 1 – 12 months of ischemic or haemorrhagic stroke
3. First onset resulting in hemiparesis of a limb with Brunnstrom Recovery Stage (BRS)

of 3, 4 or 5 (Naghdi et al. 2010)

4. Modified Ashworth Scale (MAS) with scores of less than or equal to level 2 for elbow, wrist, and hand muscle tone in the affected upper extremity (Harb & Kishner 2022)

5. Able to understand instruction

Exclusion criteria:

1. Serious cardiovascular or respiratory diseases
2. Skin lesions, infections or other skin problems
3. Serious circulatory problems such as deep vein thrombosis
4. Allergies or significant discomfort to pressure garment materials
5. Upper extremity fractures or severe joint problems
6. Severe cognitive impairment with Saint Louis University Mental Status (SLUMs) scores equal to or less than 27 (above high school level of education)/25 (below high school level of education).

9.1.2.2 Sampling Method

Participants will be recruited through convenience sampling. Convenience sampling is a common sampling method whose core features are convenience and economy (Sedgwick 2013). When conducting convenience sampling, the researcher selects those samples that are easily accessible, rather than obtaining samples through random selection. This means that the sample is usually chosen based on the convenience of the researcher, such as choosing volunteers or those who are easily accessible (Stratton 2021). It can help the researcher to obtain data quickly, but potential biases and limitations must be carefully considered when analysing and interpreting the results (Etikan et al. 2016).

9.1.2.3 Sample Size Calculation

Sample size was calculated using G*Power 3.1. Assuming that the data is normally distributed, this study will use mixed model ANOVA to compare differences between

the effects of DD-10, S-10, and S-0 pressure garment on sensorimotor functions and quality of life among stroke patients at three different time points during the 8 weeks of application. With small to medium effect size, f of 0.125, power of 0.8, α error of 0.05, three groups, and three measurements, the total sample size needed will be 132, as shown in Figure 4. With 44 people in each group and taking into account the 20 % dropout rate, by using the formula: N= n/(1-d), i.e., 44/(1-0.2) = 55, there will be a total of 55 people in each group. Thus, the final sample size will be 165 patients (n: DD-10 Group = 55; S-10 Group = 55; S-0 Group = 55).

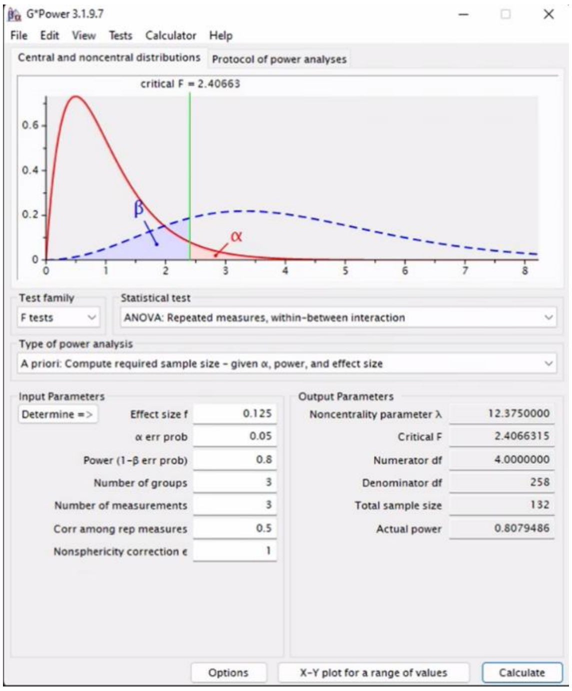

Figure 4. Sample Size Calculation

9.1.3 Research Location

This study will be conducted within the rehabilitation departments of three hospitals affiliated with Shandong Second Medical University, Weifang City, Shandong Province, China. These hospitals are the Affiliated Hospital of Shandong Second Medical University, Weifang Municipal People's Hospital, and Weifang Hospital of Traditional Chinese Medicine. These hospitals have equal medical and rehabilitation facilities and expertise, including the occupational therapy services.

The Rehabilitation Department of Weifang Hospital of Traditional Chinese

Medicine was established in 1997, operates 50 beds, 10 rehabilitation doctors, 20 rehabilitation therapists, and opening sections of physiotherapy, occupational therapy, etc. Over the years, the staff of the whole department has made unremitting explorations on the diagnosis and treatment of cerebrovascular disease, post-surgery of brain tumor, cerebral palsy, etc., and especially accumulated rich experience in the treatment of limb disorders caused by neurological injuries.

The Rehabilitation Department of Affiliated Hospital of Weifang Medical College is one of the earliest departments to carry out rehabilitation treatment in Weifang City. It currently operates 35 beds and departments of occupational therapy and physiotherapy, etc. It has 12 rehabilitation physicians and 14 rehabilitation therapists. Rich in experience of neurological rehabilitation, the hospital is equipped with advanced rehabilitation equipment, capable to carry out the tasks of treating more than 100 patients.

The Department of Rehabilitation of Weifang People's Hospital is a key clinical specialist facility in Weifang City, which currently operates 36 beds and both departments of occupational therapy and physical therapy. The hospital has nine rehabilitation physicians and 15 rehabilitation therapists to carry out comprehensive rehabilitative and medical works, including neurological rehabilitation, spinal cord injury rehabilitation, and post-surgical orthopaedic rehabilitation. The hospital is equipped with a complete set of modern assessment and rehabilitation therapy equipment.

9.1.4 Research Instruments

9.1.4.1 Pressure Garment

The pressure garments used in this study will be made and supplied by Klarity, China, with a Lycra content of 45 % and a polyamide content of 55 %.

9.1.4.1.1 Determination of Compression Pressure

Determination of compression pressure will be done using the Reduction Factor method

(Macintyre & Ferguson 2013), which is generally expressed as a percentage of stress (n%) depending on the tension generated by the pressure garment used. Different parts of the body, people, and styles of pressure garment require different percentages of stress. A typical pressure garment takes a value of 10% to 20% (Syron-Jones & Macintyre 2022). Considering that stroke patients do not need as much pressure as burn patients to inhibit scarring, we decided to use 10% stress. To achieve the predetermined stress, the dimensions required for fabrication of the take-up should be smaller than the actual measurements, the relationship of which can be expressed by the following equation:

$$L = X/ (1 + 10\%)$$

X: actual measured value; L: calculated value required to produce a certain stress (Pratt & West 1995).

9.1.4.1.2 Single-Layered 10% Circumferential Reduction (S-10) Pressure Garment

The overall design of the single-layer pressure garment is similar to that of the anti-scarring pressure garment for burn patients. It is divided into the following main steps:

1. Making the compression arm sleeve.

(a) Measuring

(b) Drawing paper pattern

(c) Cutting and sewing.

2. Fabrication of the compression glove:

(a) Measurement of the fingers in the extended position, with each finger extended and abducted, with a soft ruler and recording the values.

(b) Drawing of sample paper

(c) Drawing of paper pattern for thumb

(d) Cutting and sewing

Following these steps, the final finished single layer pressure garment is shown in Figure 5.

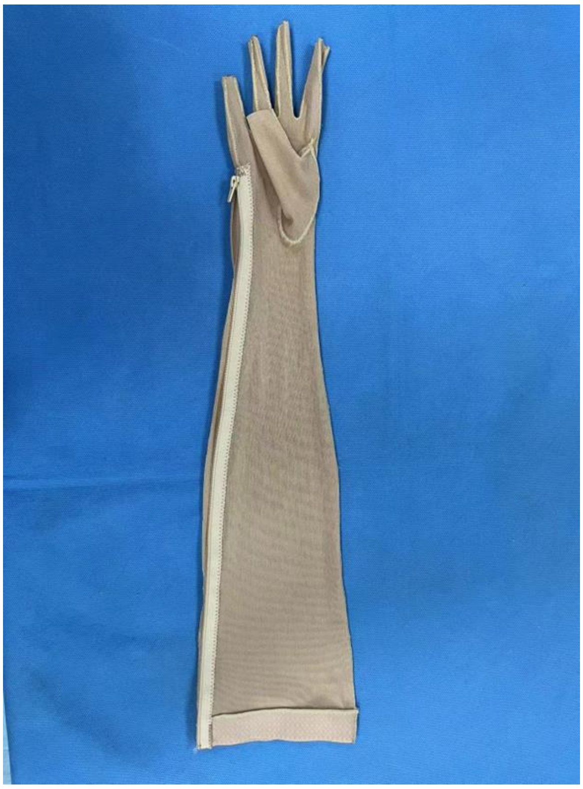

Figure 5. Single-layered 10% circumferential reduction (S-10) pressure garment

9.1.4.1.3 Dorsal Double-Layered 10% Circumferential Reduction (DD-10) Pressure Garment

The overall approach to the DD-10 pressure garment is the same as that for the S-10 pressure garment with the main difference being that the dorsal side is simply secured using two pieces of fabrics of the same size, and then sewn together as a whole. The treatment on the thumb is simplified accordingly by not hollowing it out and measuring it along with the thumb when making the paper pattern, as shown in the Figure 6.

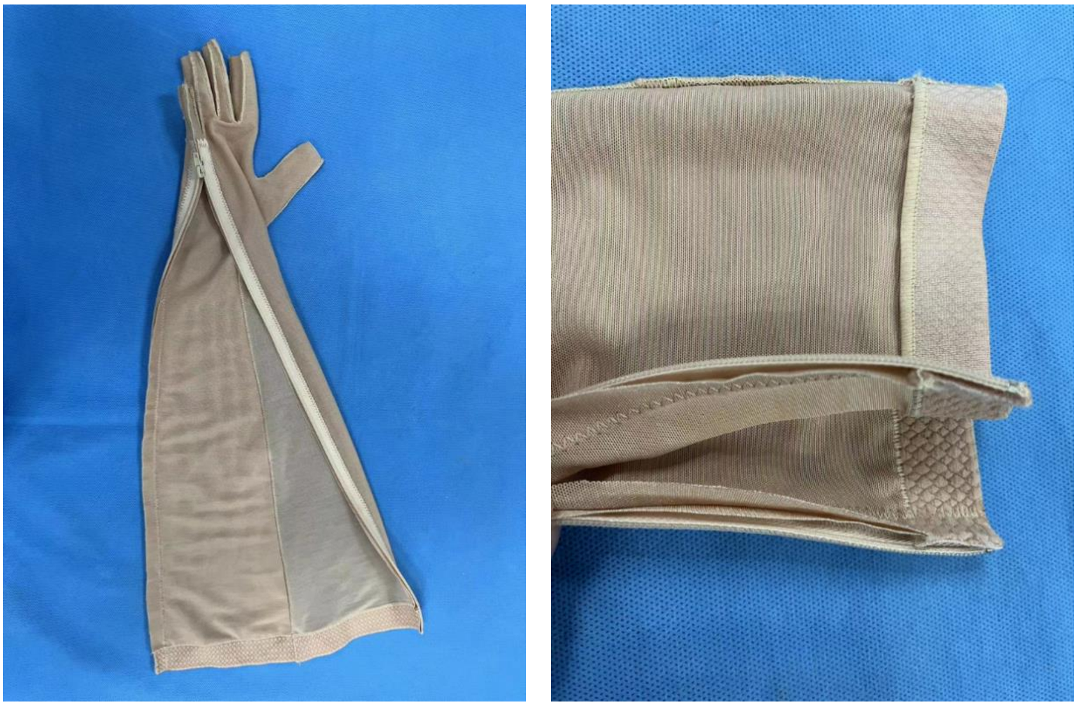

Figure 6. Dorsal double-layered (DD-10) pressure garment

9.1.4.1.4 Single-Layered No Circumferential Reduction (S-0) Pressure Garment

S-0 is pressure-free pressure garment designed to be used as a control/placebo for this study. S-0 involves mainly the same fabrication process as SS-10 except it takes the value of 0 in the ratio of reduction. Thus, participants the control group will not feel constricted/compressed while wearing this pressure garment, as shown in the Figure 7.

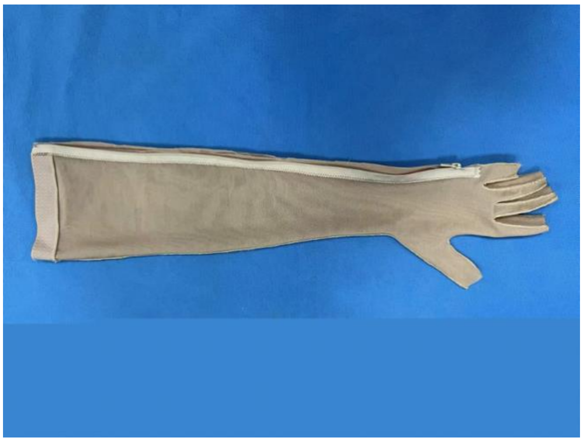

Figure 7 Single-layered no circumferential reduction (S-0) pressure garment

Considering that pressure garment need to be washed regularly to ensure cleanliness and hygiene, and to prevent the growth of bacteria, each participant in this study will

be provided with two sets of identical pressure garment for easy replacement and cleaning.

9.1.4.2 Demographic Form (Participant) (Appendix A)

The Demographic Form (Participant) is designed to collect personal, family, and medical information related to stroke patients. This form contains five sections. The first section includes personal information, including name, age, gender, and contact information. The second section focuses on medical information, including type of stroke and sequelae. The third section is on lifestyle and social background, including marital status, education level, occupation, smoking history, and drinking history. The fourth section is on family status, including numbers of family members and caregivers. Finally, the fifth section allows participants to provide any additional comments or information.

9.1.4.3 Assessment Instruments

The assessment instruments include Fugl-Mayer Assessment of Upper Extremity as a primary outcome measure; Box and Block Test, Modified Ashworth Scale, Visual Analogue Scale, and Disabilities of Arm, Shoulder and Hand as secondary outcome measures; and Brunnstrom Recovery Stage and Saint Louis University Mental Status as screening measures.

9.1.4.3.1 Fugl-Meyer Assessment of Upper Extremity (Appendix B) – Primary Outcome Measure

The Fugl-Meyer Assessment (Kaufman et al.) of Upper Extremity is a classic tool used to assess the rehabilitation process of stroke patients. The assessment is known for its comprehensiveness and standardization and is designed to provide insight into the patient's motor function and level of life activities (Deakin et al. 2003). It has two main domains, the upper extremity and the lower extremity for systematically measure the patient's motor abilities. The upper extremity domain includes elements such as range

of motion of the shoulder, elbow, wrist and fingers, as well as grip strength and hand coordination. The lower extremity domain, on the other hand, focuses on hip, knee, and ankle mobility, as well as lower extremity muscle coordination and the patient's balance (Deakin et al. 2003). Aligning with the objectives of this study, only the upper extremity domain will be used.

The Fugl-Meyer assessment had high reliability with Interclass Correlation Coefficient (ICC) = 0.79 (Carmona et al. 2023) and excellent validity with r = 0.74-0.93 (P < 0.0001) (Jill See et al. 2013). The Chinese version had good reliability with ICC = 0.988 on test-retest and ICC = 0.959 on inter-rater (Kou et al. 2013). Its validity was highly correlated with Action Arm Research Test (ARAT) at 2 weeks (r = 0.91) and at 8 weeks post-stroke (r = 0.94), P < 0.01 (Gladstone DJ et al. 2002).

9.1.4.3.2 Visual Analogue Scale (Appendix C) – Secondary Outcome Measure

The Visual Analogue Scale (Venckūnas et al.) is a scaling tool for subjective measures, often used to assess how an individual feels or sensed in a particular dimension (Shafshak & Elnemr 2021). It is a continuous scale that allows the person being assessed to indicate the degree of their subjective feelings by choosing a point between two extremes (Faiz 2014). The VAS is often used for pain assessment, assessment of emotional states, and other measures of subjective feelings. Typically, the two endpoints of the VAS are represented by extreme descriptions or emoticons. For example, in a pain assessment, one end of the scale might be "no pain" and the other end " worst pain " (Dourado et al. 2021). The person being assessed is asked to choose a point between the two endpoints that reflects their subjective feelings on that dimension. This can be done by drawing a vertical line, placing a marker, or other means (Reich et al. 2012).

The English version of the VAS had good reliability and validity, ICC = 0,97 (Bijur et al. 2001). It was strongly correlated with Numeric Rating Scale (NRS) with r = 0.92 (P < 0.001) (Shafshak & Elnemr 2021). The Chinese version also had good reliability, ICC = 0.73 and in terms of validity, it was strongly correlated with NRS,

Verbal Descriptor Scale (VDS), and the Faces Pain Scale Revised (FPS-R) (r = 0.71-0.99) (Li et al. 2007).

9.1.4.3.3 Disabilities of Arm, Shoulder and Hand Outcome Measure (Appendix D) – Secondary Outcome Measure

The Disabilities of Arm, Shoulder and Hand (DASH) Outcome Measure is a widely used measurement tool for assessing upper extremity function. It provides a comprehensive and reliable way to assess the outcome of patients with dysfunctions of the arm, shoulder and hand, particularly in conditions such as trauma, surgery or chronic pathology (Hudak et al. 1996). The DASH measure consists of a 30-items that covers a wide range of functional problems that patients face in their daily lives. These questions cover a wide range of aspects, including life activities, symptoms, social roles and psychological aspects (Beaton, Katz, et al. 2001). Patients are asked to answer the questions based on their own experience, and a score is used to reflect the degree of dysfunction in different areas, with a score of 0 indicating no dysfunction and a score of 100 indicating the most severe dysfunction (Beaton, Davis, et al. 2001).

The English version of DASH had high reliability, ICC = 0.84-0.97 (Sigirtmac & Oksuz 2021). It is strongly correlated with NDII Score (r = -0.86) (Goldstein et al. 2015). The Simplified Chinese version of the DASH, translated by Dr. Le Qi, available via DASH official website will be used in this study.

Permission to use the simplified Chinese version has been obtained from the author of this translated version, China-Japan Union Hospital of Jilin University (Appendix D2).

9.1.4.3.4 Box and Block Test (Appendix E)– Secondary Outcome Measure

The Box and Block Test (BBT) is a quantitative tool commonly used to assess upper extremity sensorimotor function. The test is designed to measure extremity coordination, dexterity, speed of movement, fine motor skills. It is a simple but effective test that is widely used in the field of rehabilitation, clinical practice, and scientific research. BBT requires the patient to move a set of cubes from one side of a box

separated into two equal-sized compartments to the other side, with a divider to prevent the cubes from falling out (Oliveira et al. 2016). BBT is assessed based on the number of cubes the patient successfully transfers in 1 minute. Typically, the faster the patient and the greater the number of blocks transferred, the better the upper extremity dexterity is considered to be. This makes the BBT an easy and objective measure (Chanubol et al. 2012), as shown in Fig. 8.

The reliability of the English version of the BBT is also relatively high, with ICC= 0.92-0.97, correlated the arm function test (r = 0.59) (Desrosiers et al. 1994). The reliability of the Chinese version using affected hand ICC= 0.975, using un-affected hand ICC= 0.982 (Li et al. 2020). The validity is also better (Li et al. 2020).

9.1.4.3.5 Modified Ashworth Scale (Appendix F) – Screening and Secondary Outcome Measure

The Ashworth Score is a measure for assessing muscle tone and is commonly used in the assessment of patients with neurological disorders, particularly when assessing patients with spastic paralysis (Lee et al. 1989). The Modified Ashworth Score (MAS) is a modification of the Ashworth score. It is also a method used to assess muscle tone or muscle spasticity, and is primarily used in patients with central neurological disorders, especially stroke patients (Harb & Kishner 2022). It is more detailed and systematic in its assessment of muscle spasticity than the traditional Ashworth score and still uses a scale of 0 to 4, where 0 indicates no spasticity and 4 indicates severe spasticity, with each scale being clearly described (Blackburn et al. 2002).

The reliability and validity of the Modified Ashworth English version was relatively high with ICC=0.874-0.973 (Akpinar et al. 2017). The Chinese version had ICC=0.58-0.89 and highly correlated with BI (Barthel Index) (r=0.816 to 1.000, p<0.01) & with the motor component of Functional Independence Measure (M-FIM) total score (r= 0.935 to 0.981, p<0.01), indicating good reliability and validity (Li et al. 2014).

9.1.4.3.6 36-Item Short Form Health Survey (Appendix G) – Secondary Outcome

Measure

The 36-Item Short Form Health Survey (SF-36) is a 36-item questionnaire designed to provide insight into a patient's health status and quality of life. Not only does this questionnaire cover a number of areas in a concise format, but the items are designed to provide a comprehensive assessment of the physical, psychological, and social dimensions of the patients (Patel et al. 2007). It includes aspects of physical functioning, limitations of physical role, social functioning, pain, and mental health (Laucis et al. 2015), etc.

The English version of SF-36 has ICC=0.96, associated with the Mental Component Summary MCS ($r=0.72$, $p<0.01$), showing relatively good reliability and validity (Russo et al. 1998). The Chinese version of the SF-36 was translated by Li et al. (2003). The Chinese version has Cronbach's $\alpha=0.791$, and structural validity, $r>0.5$, showing good reliability and validity (Zhang et al. 2012).

9.1.4.3.7 Brunnstrom Recovery Stage (BRS) (Appendix H) – Screening Outcome Measure

The Brunnstrom Recovery Stage (BRS) is a brief and easy-to-administer measure used to assess motor function. BRS consists of three items for the arm (BRS-A), hand (BRS-H), and leg (BRS-L), all of which are scored on a 6-point Likert-type scale, with each stage representing a different motor characteristic and change in muscle control (Shah et al. 1986). These items are usually used individually to describe the patient's motor function (i.e., arm, hand, and leg, respectively).

The reliability and validity of the English version of the BRS were relatively good, in which it was highly correlated to the Modified Modified Ashworth Scale (MMAS) scores ($r = 0.81$, $p < 0.0001$) (Naghdi et al. 2010). The Rasch reliabilities of the upper-extremity items and overall motor items were high (0.91–0.92) (Huang et al. 2016).

9.1.4.3.8 Saint Louis University Mental Status (SLUMS) Examination (Appendix I) – Screening Outcome Measure

The Saint Louis University Mental Status (SLUMS) Examination is a measure used to

assess cognitive functioning, specifically for early detection and monitoring of mild cognitive impairment and early stages of dementia (Tariq et al. 2006). SLUMS includes assessments of several cognitive domains such as attention, memory, executive functioning, orientation, and numeracy (Cao et al. 2012). Specific items include vocabulary learning, memory recall, numeracy, clock drawing, number reversal, and image naming (Shwartz et al. 2019). It is scored on a 30-point scale, with higher scores indicating better cognitive functioning and lower scores suggesting impaired cognitive functioning. Items are designed to cover multiple cognitive domains to provide a more comprehensive assessment.

The English version of SLUMs has ICC=0.990-0.998 and correlated with Montreal Cognitive Assessment (MoCA) score ($r=0.831, p<0.0001$), showing good reliability and validity (Noyes et al. 2023). (Naghdi et al. 2010). The Chinese version of the SLUMS was translated by Zhang et al. (2021). The Chinese version had Cronbach's $\alpha=0.723$, ICC=0.990-0.998 ($p<0.05$), at a score of 23.5, the sensitivity & specificity were 0.738 & 0.725, respectively, showing that that the reliability and validity were relatively good (Zhang et al. 2021).

Permission to use the simplified Chinese version has been obtained from the author of this translated version, Third Hospital of Soochow University (Appendix J2).

9.1.4.4 Pressure Garment Application Form (Appendix J)

The Pressure Garment Application Form is a self-developed form that includes items pertaining to pressure garment application including wearing time of pressure garment, frequency and length of occupational therapy session, and caregiver participation, etc. This form will be filled out by the participants or their family member on a daily basis and will be supervised by their therapists.

9.1.5 Data Collection

Participants will be asked to read the Phase I information sheet (Appendix A) and sign the written informed consent form (Appendix L) before participating in this phase of

study.

This study will involve Therapists A and B (n: Therapist A=3; Therapist B=6) from all three participating hospitals. Therapists selected will need to have a bachelor's degree or above, and with minimum of three years of relevant work experience. The therapists will be labelled as: Therapist A, who will serve as an assessor and blinded; Therapist B, who will serve as the participant's treating therapist and blinded. The study will also include a pressure garment fabricator. The fabricator (a therapist, a researcher, or a professional tailor) must have experience in pressure garment fabrication.

Prior to the data collection, all the therapists will receive an 8-hour training on pressure garment from the pressure garment fabrication expert, whom is one of the supervisory team members. The tentative training programme is shown below:

Time	Content
08:30 – 09:00	Registration and welcome; Introduction to the purpose of the training and programme
09:00 – 10:00	Basics of pressure garment
10:00 – 10:15	Coffee break
10:15 – 12:15	Pressure garment measurement and pattern making I
12:15 – 12:45	Education on care of pressure garment care
12:45 – 13:45	Lunch break
13:45 – 14:30	Pressure garment measurement and pattern making II
14:30 – 15:00	Putting on and adjusting pressure garment
15:00 – 15:15	Coffee break
15:15 – 16:30	Simulated practical session and group exercise
16:30 – 17:00	Programme summary
17:00 – 17:30	Q&A

For randomization purposes, the researcher will generate 165 sequences that contains equal numbers of 1 (S-10), 2 (DD-10), and 3 (S-0) using a computerized software SPSS 26.0. The sequence will be store electronically. The process of data

collection will then proceed with participant recruitment. This recruitment will be done by placing posters that contain the relevant information of this study, including the study objectives, procedures, and duration alongside contact information, etc. in the three hospitals as permitted. Interested patients can contact the researcher of this study. Recruitment will also be done through recommendation made by therapists who are involved in treating stroke patients. Before enrolling into the study, potential participants will be screened using MAS, SLUMS, and BRS by Therapist A. Only those who meet the inclusion will be enrolled. Prior to the enrolment, the potential participants will need to read the information sheet (Appendix L) and sign the written informed consent form (Appendix M). Participants will be asked to complete the Demographic Form (Participant) (Appendix A).

After participant enrolment, Therapist A will report to the researcher to obtain the randomization sequence number generated using SPSS 26.0. The researcher will assign the participant into either S-10 (intervention group), DD-10 (intervention group), or S-0 (placebo group) according to the sequence list. Therapist A, a blinded therapist will then proceed with taking the baseline data or Time 1 (start of trial), including FMA, BBT, MAS, VAS, DASH, and SF-36. After that, Therapist A will take the pressure garment measurement and then, give the pressure garment measurement to the pressure garment fabricator for pressure garment custom fabrication. Upon receiving the two pairs of pressure garment (within 7 days after pressure garment measurement), the participant will try it on for 30 minutes, under the supervision of Therapist B, a blinded therapist. The participant will formally be enrolled in the study if he/she does not experience allergy, oedema, pain or any other discomfort. All participants will be asked to wear the provided pressure garment for 3 hours in the morning, 3 hours in the afternoon, and 8 hours at night each day for 8 weeks.

During Phase IA, i.e., the first four weeks between Time 1 (Baseline) and Time 2 (Week 4), when participants are hospitalized in the rehabilitation department of the hospital, he/she will be required to attend an occupational therapy session conducted by Therapist B once a day for 30 minutes, 5 days a week. During Phase IB, i.e., the second

four weeks between Time 2 (Week 4), and Time 3 (Week 8), when participants were being discharged from the hospital. Although they need to continually wearing the pressure garment according to same wearing schedule, they will not need to attend occupation therapy session.

Each participant will be given a Pressure Garment Application Form (Appendix K) to complete. The form will need to be returned to the researcher during Time 2 (Week 4) assessment. A new form will be given for the participant to complete and collected by the researcher during Time 3 (Week 8) assessment. Therapist B will constantly monitor the condition of the pressure garment to make sure it stays tightly fit throughout the study. Depends on the garment condition, torn or loosed pressure garment will be repaired or replaced using a new pair of garments.

For Time 2 (Week 4) and Time 3 (Week 8) assessments, the FMA, MAS, BBT, and VAS will be performed by Therapist A. The DASH and SF-36 will be administered by Therapist A and completed by the participant; however, if needed, it can be completed with the help of a caregiver or a therapist. The MAS will be assessed immediately after removal of the pressure garment, and the DASH, and SF-36 will be completed within 30 minutes of removal. The order of assessments will be determined by drawing a ballot. The FMA, BBT, VAS will be assessed 30 minutes after removal and the order of assessment will also be determined by drawing a ballot.

Phase IA = Pressure Garment + Occupational Therapy for 4 weeks

Phase IB = Pressure Garment for 4 weeks

Therapist B, a treating therapist will provide a list of specific occupational therapy interventions, identified in this study as representing routine practice to participants. Therapist B will record the Pressure Garment Application Form (Appendix K). These specific occupational therapy interventions will be given according to the following sequence:

1. Warm-up activities for 5 minutes, targeting on increasing body temperature, improve blood circulation, and prepare the body to participate in subsequent therapeutic activities. Activities used will include easy upper extremity exercises such as arm

swings, finger flexion and extension and wrist activities.

2. Remedial activities for 10 minutes, targeting on improving specific motor, strength, or coordination skills in response to the participant's specific rehabilitation goals. Activities used will include building blocks, moving sticks, puzzles, twisting screws, threading beads, etc.

3. Activities of daily living (Harrell & Bradley) training for 10 minutes, targeting on improving ability to perform basic daily living activities independently in daily life. Activities used include simulated daily life scenarios for actual ADL training (e.g. bathing, dressing, eating, etc.), use of assistive devices and step-by-step guidance to ensure participant’s safety and successful completion.

4. Relaxation activities for 5 minutes, targeting on relieving tension and anxiety. Activities used will include deep breathing exercises, progressive muscle relaxation that focuses on stress-prone areas such as the neck, shoulders and back, static stretches and other exercises to help relax the body.

During the Time 3 (Week 8) assessment, the success of blinding assessment using the statistical method of James’ Blinding Index (BI) (James et al. 1996) will be conducted. All participants will be asked to indicate the group that they belonged to, i.e., either DD-10, S-10, S-0 or don’t know (Appendix N). Therapist A who is blinded will also be asked to indicate the same question regarding group location of the participant that they had assessed (Appendix O). Those who do not answer the questions will be removed from the blinded assessment.

James et al. (1996) proposed a BI, as a variation of the kappa coefficient, that is sensitive not to the degree of agreement but to the degree of disagreement, by placing the highest weight on ‘do not know’ responses. This index ranges from 0 to 1, 0 being total lack of blinding, 1 being complete blinding and 0.5 being completely random blinding (i.e. 50% correct and 50% incorrect guesses) (James et al. 1996). If the upper bound of the confidence interval (CI) of BI is below 0.5 (i.e., CI does not cover the null value), the study is regarded as lacking blinding (James et al. 1996). Otherwise, one may conclude that there is insufficient evidence for unblinding.

9.1.6 Data Analysis

Data will be analysed using SPSS 26.0 using Intention-to-treat (ITT) analysis. ITT analysis, which aims to minimize bias and maintain random allocation of study participants (Gupta 2011), is suitable to be used when there are shedding participants. Its key idea is to analyse the group to which the participant belongs in that initial allocation, regardless of whether they completed the study or received the intended intervention. In clinical trials, participants may drop out, refuse intervention, or deviate from the study protocol. The ITT analysis includes all participants regardless of whether they completed the study or followed the treatment plan. This helps prevent shedding bias, which may occur if only completers are analysed (Mccoy 2017). The study will use a imputation method, such as, Last Observation Carried Forward (LOCF) (Shao & Zhong 2003) or mean imputation (Jadhav et al. 2019), or multiple imputation (Austin et al. 2021), this will probably depend on the requirements of the data.

Both descriptive and inferential statistics will be used. Frequencies, means, and medians will be used to describe the demographic data (Fisher & Marshall 2009). A mixed model ANOVA to compare the outcome measures.

9.1.7 SPIRIT Process

SPIRIT (Standard Protocol Items: Recommendations for Interventional Trials) is a guidance document designed to provide transparency of research protocols, particularly for interventional trials. SPIRIT's goal is to ensure that trial protocols are clear, detailed, and comprehensive in order to promote the scientific validity and replicability of the study (Chan et al. 2013). The use of SPIRIT helps to ensure that trial protocols are transparent, providing investigators, reviewers, and other stakeholders with comprehensive information to enable them to understand the design and execution of the trial. This contributes to the scientific, replicable, and ethical nature of the trial (Chan et al. 2015). The SPIRIT process that will be used in this study is shown in Figure 8.

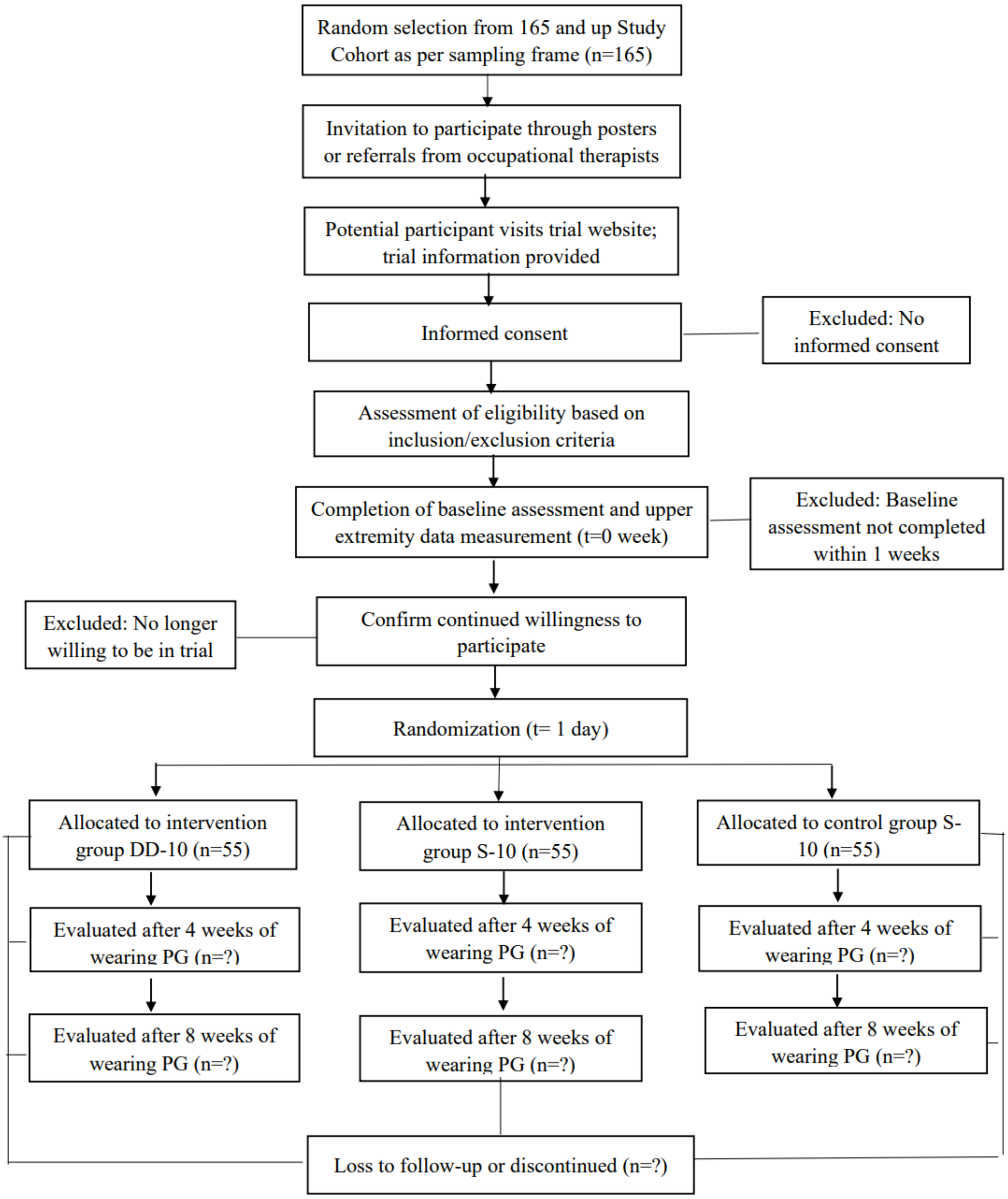

Figure 8. SPIRIT Process

10.0 Reference

Akpinar, P., Atici, A., Ozkan, F., Aktas, I., Kulcu, D., Sarı, A. & Durmus, B. 2017. Reliability of the Modified Ashworth Scale and Modified Tardieu Scale in patients with spinal cord injuries. *Spinal Cord* 55(10): 944-949.

Anzarut, A., Olson, J., Singh, P., Rowe, B. H. & Tredget, E. E. 2009. The effectiveness of pressure garment therapy for the prevention of abnormal scarring after burn injury: a meta-analysis. *J Plast Reconstr Aesthet Surg* 62(1): 77-84.

Austin, P. C., White, I. R., Lee, D. S. & Van Buuren, S. 2021. Missing data in clinical research: a

tutorial on multiple imputation. *Canadian Journal of Cardiology* 37(9): 1322-1331.

Beaton, D. E., Davis, A. M., Hudak, P. & Mcconnell, S. 2001. The DASH (Disabilities of the Arm, Shoulder and Hand) outcome measure: what do we know about it now? *The British Journal of Hand Therapy* 6(4): 109-118.

Beaton, D. E., Katz, J. N., Fossel, A. H., Wright, J. G., Tarasuk, V. & Bombardier, C. 2001. Measuring the whole or the parts?: validity, reliability, and responsiveness of the Disabilities of the Arm, Shoulder and Hand outcome measure in different regions of the upper extremity. *Journal of Hand Therapy* 14(2): 128-142.

Béjot, Y., Daubail, B. & Giroud, M. 2016. Epidemiology of stroke and transient ischemic attacks: Current knowledge and perspectives. *Rev Neurol (Paris)* 172(1): 59-68.

Bijur, P. E., Silver, W. & Gallagher, E. J. 2001. Reliability of the visual analog scale for measurement of acute pain. *Academic emergency medicine* 8(12): 1153-1157.

Blackburn, M., Van Vliet, P. & Mockett, S. P. 2002. Reliability of measurements obtained with the modified Ashworth scale in the lower extremities of people with stroke. *Phys Ther* 82(1): 25-34.

Cao, L., Hai, S., Lin, X., Shu, D., Wang, S., Yue, J., Liu, G. & Dong, B. 2012. Comparison of the Saint Louis University Mental Status Examination, the Mini-Mental State Examination, and the Montreal Cognitive Assessment in detection of cognitive impairment in Chinese elderly from the geriatric department. *Journal of the American Medical Directors Association* 13(7): 626-629.

Carmona, C., Sullivan, J. E., Arceo, R., Drogos, J., Besser, S., Gutierrez, S., Jeteric, Z., Wyman, J. & Yao, J. 2023. Development and preliminary validity study of a modified version of the upper extremity Fugl-Meyer assessment for use in telerehabilitation. *Journal of Neurologic Physical Therapy* 47(4): 208-216.

Carod-Artal, F. J. & Egido, J. A. 2009. Quality of life after stroke: the importance of a good recovery. *Cerebrovascular diseases* 27(Suppl. 1): 204-214.

Chan, A.-W., Tetzlaff, J. M., Altman, D. G., Laupacis, A., Gøtzsche, P. C., Krleža-Jerić, K., Hrobjartsson, A., Mann, H., Dickersin, K. & Berlin, J. A. 2015. SPIRIT 2013 Statement: defining standard protocol items for clinical trials. *Revista Panamericana de Salud Pública* 38: 506-514.

Chan, A.-W., Tetzlaff, J. M., Altman, D. G., Laupacis, A., Gøtzsche, P. C., Krleža-Jerić, K., Hróbjartsson, A., Mann, H., Dickersin, K. & Berlin, J. A. 2013. SPIRIT 2013 statement: defining standard protocol items for clinical trials. *Annals of internal medicine* 158(3): 200-207.

Chanubol, R., Wongphaet, P., Chavanich, N., Chira-Adisai, W., Kuptniratsaikul, P. & Jitpraphai, C. 2012. Correlation between the action research arm test and the box and block test of upper extremity function in stroke patients. *Journal of the Medical Association of Thailand* 95(4): 590.

Deakin, A., Hill, H. & Pomeroy, V. M. 2003. Rough guide to the Fugl-Meyer Assessment: upper limb section. *Physiotherapy* 89(12): 751-763.

Desrosiers, J., Bravo, G., Hébert, R., Dutil, É. & Mercier, L. 1994. Validation of the Box and Block Test as a measure of dexterity of elderly people: reliability, validity, and norms studies. *Arch Phys Med Rehabil* 75(7): 751-755.

Dourado, G. B., Volpato, G. H., De Almeida-Pedrin, R. R., Pedron Oltramari, P. V., Freire Fernandes, T. M. & De Castro Ferreira Conti, A. C. 2021. Likert scale vs visual analog scale for assessing facial pleasantness. *Am J Orthod Dentofacial Orthop* 160(6): 844-852.

Etikan, I., Musa, S. A. & Alkassim, R. S. 2016. Comparison of convenience sampling and purposive sampling. *American journal of theoretical and applied statistics* 5(1): 1-4.

Faiz, K. W. 2014. [VAS--visual analog scale]. *Tidsskr Nor Laegeforen* 134(3): 323.

Feigin, V. L., Brainin, M., Norrving, B., Martins, S., Sacco, R. L., Hacke, W., Fisher, M., Pandian, J. & Lindsay, P. 2022. World Stroke Organization (WSO): Global Stroke Fact Sheet 2022. *Int J Stroke* 17(1): 18-29.

Fisher, M. J. & Marshall, A. P. 2009. Understanding descriptive statistics. *Australian critical care* 22(2): 93-97.

Gerard, Toussaint-Thorin, M., Mohammad, Y., Letellier, G., Fritot, S., Masson, S., Duhamel, A., Donskoff, C., Zagame, Y., Beghin, L. & Gottrand, L. 2022. PROPENSIX: pressure garment therapy using compressive dynamic Lycra(®) sleeve to improve bi-manual performance in unilateral cerebral palsy: a multicenter randomized controlled trial protocol. *Trials* 23(1): 117.

Goldstein, D. P., Ringash, J., Irish, J. C., Gilbert, R., Gullane, P., Brown, D., Xu, W., Del Bel, R., Chepeha, D. & Davis, A. M. 2015. Assessment of the Disabilities of the Arm, Shoulder, and Hand (DASH) questionnaire for use in patients after neck dissection for head and neck cancer. *Head & Neck* 37(2): 234-242.

Gupta, S. K. 2011. Intention-to-treat concept: a review. *Perspectives in clinical research* 2(3): 109.

Harb, A. & Kishner, S. 2022. Modified ashworth scale. In (ed.). *StatPearls [Internet]*: StatPearls Publishing.

Harrell, M. C. & Bradley, M. 2009. Data collection methods: Semi-structured interviews and focus groups.

Hatem, S. M., Saussez, G., Della Faille, M., Prist, V., Zhang, X., Dispa, D. & Bleyenheuft, Y. 2016. Rehabilitation of motor function after stroke: a multiple systematic review focused on techniques to stimulate upper extremity recovery. *Frontiers in human neuroscience* 10: 442.

Huang, C.-Y., Lin, G.-H., Huang, Y.-J., Song, C.-Y., Lee, Y.-C., How, M.-J., Chen, Y.-M., Hsueh, I.-P., Chen, M.-H. & Hsieh, C.-L. 2016. Improving the utility of the Brunnstrom recovery stages in patients with stroke: validation and quantification. *Medicine (Baltimore)* 95(31):

Hudak, P. L., Amadio, P. C., Bombardier, C., Beaton, D., Cole, D., Davis, A., Hawker, G., Katz, J. N., Makela, M. & Marx, R. G. 1996. Development of an upper extremity outcome measure: the DASH (disabilities of the arm, shoulder, and head). *American journal of industrial medicine* 29(6): 602-608.

Jadhav, A., Pramod, D. & Ramanathan, K. 2019. Comparison of performance of data imputation methods for numeric dataset. *Applied Artificial Intelligence* 33(10): 913-933.

James, Bloch, Lee, Kraemer & Fuller. 1996. An index for assessing blindness in a multi-centre clinical trial: disulfiram for alcohol cessation—a VA cooperative study. *Statistics in medicine* 15(13): 1421-1434.

Jill See, M., Lucy Dodakian, M. & Cathy Chou, M. 2013. A Standardized Approach to the Fugl-Meyer Assessment and Its Implications for Clinical Trials.

Kaufman, Y., Cole, P. & Hollier, L. 2009. Peripheral nerve injuries of the pediatric hand: issues in diagnosis and management. *J Craniofac Surg* 20(4): 1011-1015.

Laucis, N. C., Hays, R. D. & Bhattacharyya, T. 2015. Scoring the SF-36 in orthopaedics: a brief guide. *The Journal of bone and joint surgery. American volume* 97(19): 1628.

Lee, K.-C., Carson, L., Kinnin, E. & Patterson, V. 1989. The Ashworth scale: a reliable and reproducible method of measuring spasticity. *Journal of Neurologic Rehabilitation* 3(4): 205-209.

Li, Lin, L.-J., Chan, A.-T., Chen, C.-H., Chang, W.-M. & Cho, Y.-J. 2020. Population based norms for the box and blocks test in healthy right-handed Taiwanese adults. *biomedical journal* 43(6): 484-489.

Li, Wu, Y. & Li, X. 2014. Test-retest reliability and inter-rater reliability of the Modified Tardieu Scale and the Modified Ashworth Scale in hemiplegic patients with stroke. *Eur J Phys Rehabil Med* 50(1): 9-15.

Li, L., Liu, X. & Herr, K. 2007. Postoperative pain intensity assessment: a comparison of four scales in Chinese adults. *Pain Medicine* 8(3): 223-234.

Li, L., Wang, H. & Shen, Y. 2003. Chinese SF-36 Health Survey: translation, cultural adaptation, validation, and normalisation. *Journal of Epidemiology & Community Health* 57(4): 259-263.

Macintyre, L. & Ferguson, R. 2013. Pressure garment design tool to monitor exerted pressures. *Burns* 39(6): 1073-1082.

Mccoy, C. E. 2017. Understanding the intention-to-treat principle in randomized controlled trials. *Western Journal of Emergency Medicine* 18(6): 1075.

Naghdi, S., Ansari, N. N., Mansouri, K. & Hasson, S. 2010. A neurophysiological and clinical study of Brunnstrom recovery stages in the upper limb following stroke. *Brain Inj* 24(11): 1372-1378.

Noyes, E. T., Major, S., Wilson, A. M., Campbell, E. B., Ratcliffe, L. N. & Spencer, R. J. 2023. Reliability and factor structure of the Saint Louis University Mental Status (SLUMS) examination. *Clinical Gerontologist* 46(4): 525-531.

Oliveira, C. S., Almeida, C. S., Freias, L. C., Santana, R., Fernandes, G., Fonseca Junior, P. R. & Moura, R. C. F. 2016. Use of the Box and Block Test for the evaluation of manual dexterity in individuals with central nervous system disorders: A systematic review. *Manual Therapy, Posturology & Rehabilitation Journal* 14: 0-0.

Ooi, H. K., Chai, S. C. & Kadar, M. 2020. Effects of pressure garment on spasticity and function of the arm in the early stages after stroke: a randomized controlled trial. *Clin Rehabil* 34(4): 515-523.

Patel, A. A., Donegan, D. & Albert, T. 2007. The 36-item short form. *JAAOS-Journal of the American Academy of Orthopaedic Surgeons* 15(2): 126-134.

Pratt, J. & West, G. 1995. *Pressure garments : a manual on their design and fabrication.* 1st ed Ed.Oxford: Butterworth-Heinemann Oxford.

Raghavan. 2015. Upper limb motor impairment after stroke. *Physical Medicine and Rehabilitation Clinics* 26(4): 599-610.

Reich, A., Heisig, M., Phan, N. Q., Taneda, K., Takamori, K., Takeuchi, S., Furue, M., Blome, C., Augustin, M. & Ständer, S. 2012. Visual analogue scale: evaluation of the instrument for the assessment of pruritus. *Acta dermato-venereologica* 92(5): 497-501.

Russo, J., Trujillo, C. A., Wingerson, D., Decker, K., Ries, R., Wetzler, H. & Roy-Byrne, P. 1998. The MOS 36-Item Short Form Health Survey: reliability, validity, and preliminary findings in schizophrenic outpatients. *Medical Care*: 752-756.

Sedgwick, P. 2013. Convenience sampling. *Bmj* 347:

Shafshak & Elnemr. 2021. The visual analogue scale versus numerical rating scale in measuring pain severity and predicting disability in low back pain. *JCR: Journal of Clinical Rheumatology* 27(7): 282-285.

Shafshak, T. S. & Elnemr, R. 2021. The Visual Analogue Scale Versus Numerical Rating Scale in Measuring Pain Severity and Predicting Disability in Low Back Pain. *J Clin Rheumatol* 27(7): 282-285.

Shah, S., Harasymiw, S. & Stahl, P. 1986. Stroke rehabilitation: outcome based on Brunnstrom recovery stages. *The Occupational Therapy Journal of Research* 6(6): 365-376.

Shao, J. & Zhong, B. 2003. Last observation carry-forward and last observation analysis. *Stat Med* 22(15): 2429-2441.

Shwartz, S. K., Morris, R. D. & Penna, S. 2019. Psychometric properties of the Saint Louis University mental status examination. *Applied Neuropsychology: Adult* 26(2): 101-110.

Sigirtmac, I. C. & Oksuz, C. 2021. Systematic review of the quality of the cross-cultural adaptations of Disabilities of the Arm, Shoulder and Hand (DASH). *La Medicina del Lavoro* 112(4): 279.

Stratton, S. J. 2021. Population research: convenience sampling strategies. *Prehospital and disaster Medicine* 36(4): 373-374.

Syron-Jones, D. & Macintyre, L. 2022. Pressure by design: How to improve the consistency of pressure garments in the clinical environment and implement a simple method for gathering evidence to establish efficacy. *Burns* 48(5): 1172-1182.

Tariq, S. H., Tumosa, N., Chibnall, J. T., Perry Iii, M. H. & Morley, J. E. 2006. Comparison of the Saint Louis University mental status examination and the mini-mental state examination for detecting dementia and mild neurocognitive disorder—a pilot study. *The American journal of geriatric psychiatry* 14(11): 900-910.

Tu, W. J., Zhao, Z., Yin, P., Cao, L., Zeng, J., Chen, H., Fan, D., Fang, Q., Gao, P., Gu, Y., Tan, G., Han, J., He, L., Hu, B., Hua, Y., Kang, D., Li, H., Liu, J., Liu, Y., Lou, M., Luo, B., Pan, S., Peng, B., Ren, L., Wang, L., Wu, J., Xu, Y., Xu, Y., Yang, Y., Zhang, M., Zhang, S., Zhu, L., Zhu, Y., Li, Z., Chu, L., An, X., Wang, L., Yin, M., Li, M., Yin, L., Yan, W., Li, C., Tang, J., Zhou, M. & Wang, L. 2023. Estimated Burden of Stroke in China in 2020. *JAMA Netw Open* 6(3): e231455.

Venckūnas, T., Trinkūnas, E., Kamandulis, S., Poderys, J., Grūnovas, A. & Brazaitis, M. 2014. Effect of lower body compression garments on hemodynamics in response to running session. *ScientificWorldJournal* 2014: 353040.

Wafa, H. A., Wolfe, C. D. A., Emmett, E., Roth, G. A., Johnson, C. O. & Wang, Y. 2020. Burden of Stroke in Europe: Thirty-Year Projections of Incidence, Prevalence, Deaths, and Disability-Adjusted Life Years. *Stroke* 51(8): 2418-2427.

Zhang, Bo, Lun, Guo & Liu. 2012. The 36-item short form health survey: reliability and validity in Chinese medical students. *International journal of medical sciences* 9(7): 521.

Zhang, Wu, Y.-H., Zhang, Y., Zhang, Y. & Cheng, Y. 2021. Preliminary study of the validity and reliability of the Chinese version of the Saint Louis University Mental Status Examination (SLUMS) in detecting cognitive impairment in patients with traumatic brain injury. *Applied Neuropsychology: Adult* 28(6): 633-640.

Appendix A Demographic Form (Participant)

Participant ID:

Date:

**Demographic Form (Participant)**	
**Personal Information**	
**1. Participant ID:**	
**2. Age:**	
**3. Gender:**	**Male/Female**
**4. Hand Dominance:**	**Right/Left**
**5. Affected Hand:**	**Right/Left**
**6. Contact address:**	
**7. E-mail address:**	
**8. Telephone number:**	
**9. Emergency contact name:**	
**10. Emergency contact phone number:**	
**Medical Information**	
**1. Date of stroke:**	
**2. Type of stroke:**	**- Ischaemic stroke**
	**- Haemorrhagic stroke**
**3. Whether received pressure garment intervention:**	**Yes/No**
**4. Hours per day of occupational therapy**	
**5. Name of therapist**	
**Intervention**	
**1. Physiotherapy**	
**2. Occupational therapy**	
**3.Traditional Chinese Medicine (TCM)**	
**4.Other treatments**	
**Lifestyle and Social Background**	
**1. Marital status:**	**- Single**
	**- Married**
	**- Divorced**
	**- Widowed**
**2. Educational attainment:**	**- Primary school**

	**- Junior high school**
	**- High School**
	**- College**
	**- Undergraduate**
	**- Postgraduate and above**
**3. Occupation:**	
**4. Smoking history:**	**- Never smoked**
	**- Used to smoke, but quit**
	**- Daily smoker**
	**- Occasional smoker**
**5. Drinking history**	**- Never drink alcohol**
	**- Moderate drinking**
	**- High drinking**
**Family Status**	
**1. Number of family members:**	
**2. Whether there is a caregiver:**	**Yes/No**
**3.Name of caregiver (if applicable):**	
**4. Relationship to participant:**	
**5.Caregiver's contact information:**	
**Additional Notes**	

Appendix B Fugl-Meyer Assessment

Appendix C Visual Analogue Scale

Participant ID:
Date:

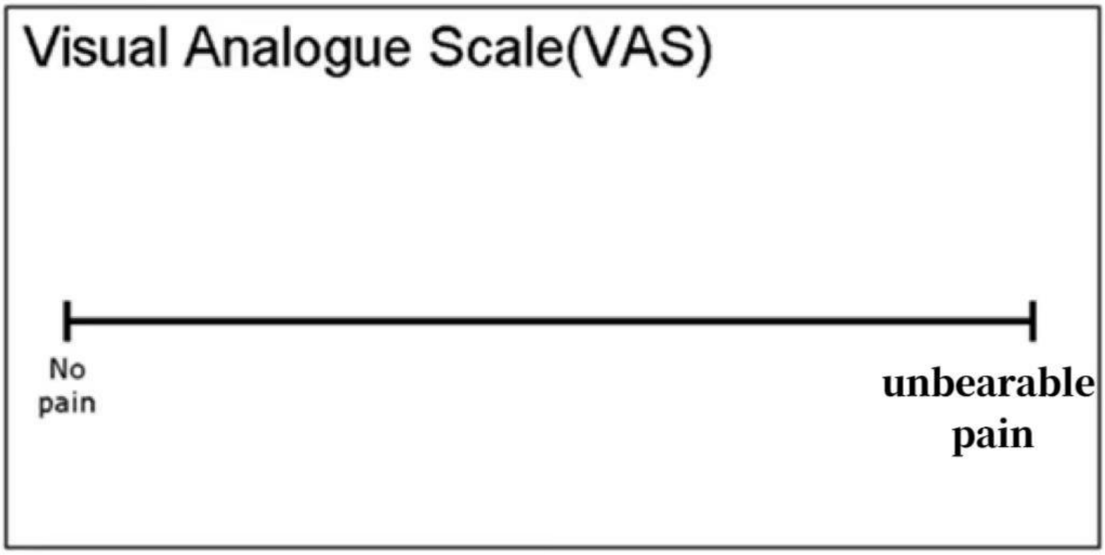

Appendix D Disabilities of the Arm, Shoulder and Hand

:

Appendix E Box and Block Test Result Form

Box and Block Test

Participant ID:
Date:

	unit (units
Left Hand	
Right hand	

Appendix F Modified Ashworth Scale

Appendix G SF-36 Questionnaire

Appendix H Brunnstrom Recovery Stage

Appendix I1 SLUMS Examination

Appendix J Pressure Garment Application Form

Participant ID:

Date:

Pressure Garment Application Form	Date	Time of use (daytime)	Time of use (night)	Treatment Frequency	Treatment Duration	Treatment Location	Name of therapist	Carer's participation	Remarks

Appendix K Information Sheet

Information Sheet

Research Title:

Effects of pressure garments of varying designs on upper extremity sensorimotor functions and quality of life after stroke: A mixed-methods study

Introduction:

We are inviting you to participate in a clinical research study. It is important that you take the time to read and understand the information in this information sheet before participating in this study.

Purpose of the Study:

Rehabilitation of upper extremity dysfunction after stroke is still a major challenge and there is little research on pressure garment intervention in post-stroke rehabilitation. Considering the good effects of pressure garment in the rehabilitation of children with cerebral palsy, including the reduction of muscle tone, we therefore would like to study the effects of different pressure garments on sensorimotor functions, motor control, dexterity, muscle tone, pain, self-perceived upper extremity function, and quality of life among stroke patients.

A total of 165 individuals like you will be participating in this study. The whole study will last about one year and your participation will be about 8 weeks only.

What is the study about?

If you agree to participate, the therapist may need to perform some tests and examinations to determine if you are suitable for the study. If you are deemed suitable, you will be randomly assigned to either one of the three groups, i.e., different intervention group or a placebo group according to randomized sequence that had already been generated. All intervention groups and placebo group will participate in an occupational therapy session for 2 hours every week for the first 4 weeks. Depends on your group allocations, you will be given two sets of pressure garments (finger-to-above-elbow- “long glove”) from us free of charge to be worn on your affected upper extremity, which you may or may not benefit from. Pressure garment will be custom fabricated to fit your fingers, hand, forearm, and arm. The pattern of your affected hand will be traced and measured. You will be constantly monitored by a therapist. Necessary adjustment will be made on the pressure garment to maintain its tight fit. You will be required to wear the pressure garment for 3 hours in the morning, 3 hours in the afternoon, and 8 hours at night for 8 weeks continuously and record the wearing time on a Pressure Garment Application Form. This form needs to be submitted to your treating therapist during reassessments at Week 4 and Week 8. The results of this study will be measured accordingly. These include:

- Motor control to be measured using Fugl-Meyer Assessment (Kaufman et al.) of Upper Extremity
- Dexterity to be measured using Box and Block Test (BBT)

- Muscle tone to be measured using Modified Ashworth Scale (MAS)
- Pain to be measured using Visual Analogue Scale (Venckūnas et al.)
- Self-perceived upper extremity function to be measured using Disabilities of Arm, Shoulder and Hand (DASH) Outcome Measure
- Quality of life to be measured using 36 Item Short Health Survey (SF-36)

All results will be taken before intervention, at 4 weeks, and at 8 weeks of the intervention.

Risks

The potential risks of participating in the study may include allergic reaction, hand become discoloured (white/blue), pain or loss of sensation in the affected upper extremity. You can remove the garment if this happens and inform the treating therapist immediately. However, these events will unlikely happens because we will only ask you to stay in the study after you shown no such events following 30 minutes of pressure garment application.

Benefits

Your participation will help us to understand how pressure garment improve your upper extremity and quality of life so that we can refine the pressure garment and improve its application for other individuals with the same condition. You will have the opportunity to see your own measurement results upon completion of this study.

Do you have to participate?

Participation in this study is voluntary. If you agree to participate, we will ask you to sign an "Informed Consent Form". You will receive a copy of this "Informed Consent" and this information sheet. If you decide not to participate in this study, you have the right to refuse. If you decide to participate, you can still withdraw from the study without penalty. If you withdraw, any data collected from you up to your withdrawal will still be used for the study. Your refusal to participate or withdrawal will not affect any medical or health benefits to which you are otherwise entitled. The researchers may also ask you to withdraw from the study for various reasons. In this case, you will not be penalised and you will not lose your patient rights.

Data and Confidentiality:

The data from this study will be produced and published. Only the research team and REC UKM will have access to the data. The data will be reported as a group and no individuals will be mentioned. Therefore, your identity will be kept confidential.

Payment and Compensation:

There is no fee to participate in this study and you will not be paid. However, you do have to pay for the usual hospital charges.

Who can I talk to about the study?
If you have any questions, you can address them directly to the research team. You can also contact REC UKM for more information.

Name: Dr. Chai Siaw Chui
Centre for Rehabilitation and Special Needs Studies
Faculty of Health Sciences
Universiti Kebangsaan Malaysia
Email: sc.chai@ukm.edu.my
Telephone (Office): 603 9289 7047

Name: Prof. Li Kuicheng
School of Rehabilitation Medicine
Shangdong Second Medical University
Email: kuichengli@163.com
Moblie:8613926144818

Name: Xu Zhenkun
PhD Candidate
Centre for Rehabilitation and Special Needs Studies
Faculty of Health Sciences
Universiti Kebangsaan Malaysia
Email: xzk1818@126.com
Mobile: 8618063595058

Appendix L Informed Consent Form

INFORMED CONSENT FORM

Research Title: Effects of pressure garments of varying designs on upper extremity sensorimotor functions and quality of life after stroke: A mixed-methods study

Researcher's Name: Xu Zhenkun,, Dr. Chai Siaw Chui, Prof. Li Kuicheng

I, ……………………………………, IC No: …………………......

- have read the information in the Information Sheet **including information regarding the risk in this study**
- have been given time to think about it and all of my questions have been answered to my satisfaction.
- understand that I may freely choose to withdraw from this study at anytime without reason and without repercussion
- understand that my anonymity will be ensured in the write-up.

I voluntarily agree to be part of this research study, to follow the study procedures, and to provide necessary information to the doctor, nurses, or other staff members, as requested.

………………………………. …………………..

(Signature) (Date)

………………………………. Witness (if any) <br>………………………………. (Signature) <br>………………………………. (IC Number) <br>…………………………..… (Date)	………………………………. Researcher <br>………………………………. (Signature) <br>………………………………. (IC Number) <br>………………………………. (Date)

Appendix M Participant blinding questionnaire

Participant ID:
Date:

Which of the following groups do you think you belong to?

A. Intervention Group DD-10

B. Intervention Group S-10

C. Placebo Group S-0

D. Don't know

Appendix N Therapist blinding questionnaire

Which of the following groups do you feel the participant you are assessing belongs to?

Participant ID:
Date:

A. Intervention Group DD-10

B. Intervention Group S-10

C. Placebo Group S-0

D. Don't know
